# Implementation of a Health Risk Assessment into Workflow of the Appointment-Based Model at an Independent Community Pharmacy

**DOI:** 10.3390/pharmacy10060148

**Published:** 2022-11-06

**Authors:** Erica Jackson, Stephanie Harriman McGrath, Joni C. Carroll, Melissa Somma McGivney, Samantha Pitzarella, Kim C. Coley

**Affiliations:** 1 Kinney Drugs, Syracuse, NY 13208, USA; 2 Pennsylvania Pharmacists Care Network, Pittsburgh, PA 15261, USA; 3 School of Pharmacy, University of Pittsburgh, Pittsburgh, PA 15261, USA; 4 Asti’s South Hills Pharmacy, Pittsburgh, PA 15234, USA

**Keywords:** health risk assessment, community pharmacy services, social determinants of health, implementation science

## Abstract

Health risk assessments (HRAs) are tools used to collect information on patients’ current health conditions, personal and family medical history, and lifestyle factors that can impact their overall health. The objectives of this pilot project were to implement an HRA as part of the appointment-based model workflow and to assess the resulting pharmacy-patient-care service opportunities. Sixteen HRA questions from a single health plan were incorporated into the appointment-based model workflow at an independent community pharmacy. Questions were administered either telephonically or in person over two patient encounters. Pharmacy staff were trained on how to administer the HRA, what to do if patients needed immediate assistance, how to provide referrals, and how to document of responses. Forty-nine patients were contacted and 38 (77.6%) completed the HRA. The median time for HRA completion was 19 min and the identified opportunities were vaccination (49), smoking cessation (15), diabetes prevention program (14), asthma control assessments (8), and substance use disorder screening and referral (3). This pilot project demonstrates that community pharmacies can implement HRAs and utilize the results to identify new pharmacy-patient-care service opportunities that can contribute to improved patient care and practice sustainability.

## 1. Introduction

Health risk assessments (HRAs) are questionnaires that assess patients for lifestyle factors and health risks that can impact their overall health. They are typically utilized by employers, health plan providers, and health care providers to gather information, such as demographics, current health conditions, personal and family medical history, and lifestyle behaviors [1]. These data can then be used at the population level to identify health and wellness initiatives or at the patient level to provide targeted services and referrals [2,3]. However, low HRA completion rates present a challenge and stakeholders are increasingly looking for new ways to engage their patients and members [4,5].

Community pharmacies are highly accessible healthcare locations with patients frequenting multiple times a year [6,7]. Patients often have trusting relationships with their pharmacist and see their pharmacist as an integral part of the health care team [8,9]. Community pharmacies are increasingly leveraging these relationships to conduct patient health screenings as well as assessments of social risk factors, with many pharmacies using the appointment-based model (ABM) to facilitate these activities [10,11,12]. By conducting HRAs with their patients, community pharmacies can address a crucial need for health plans. Additionally, the data garnered from HRAs can help pharmacy teams identify opportunities for patient care interventions (e.g., tobacco cessation and immunizations) and contribute to the sustainability of their practice. The objectives of this pilot project were to (1) implement an HRA as part of the appointment-based model (ABM) workflow at an independent community pharmacy and (2) assess pharmacy-patient-care service opportunities resulting from the HRA intervention.

## 2. Materials and Methods

This project was conducted at an independent community pharmacy, Asti’s South Hills Pharmacy, located in Pittsburgh, Pennsylvania. Asti’s South Hills Pharmacy is a high-volume pharmacy that offers enhanced patient care services, such as medication synchronization, comprehensive medication reviews, and point-of-care testing. The pharmacy is a member of the Pennsylvania Pharmacist Care Network (PPCN), an enhanced pharmacy services network of over 180 community pharmacies in Pennsylvania [13]. PPCN, in collaboration with a regional health plan, approached the pharmacy to support a pilot program aimed at the completion of HRAs in their Medicaid population. The pharmacy did not have any direct relationship with this payor prior to being approached by PPCN about their interest in this project. The project was led by a community-based pharmacy resident.

### 2.1. Development of the Pilot Program

This project was guided by the implementation stages of the active implementation frameworks [14]. The implementation stages include exploration (to assess program feasibility and team readiness), installation (to prepare for program delivery), initial implementation (to integrate program into practice), and full implementation (to integrate new learnings into future practice). Only the first three stages were applied as part of this short-term pilot project.

For the exploration stage, the project team reviewed the pharmacy’s ABM workflow and met with pharmacy team members to identify how to implement the HRA most efficiently into the ABM. Two ongoing patient care programs were identified as implementation points for the HRA. These were the pharmacy’s medication synchronization program, where patients receive a monthly phone call from the pharmacy after their medications are ready for pickup or delivery, and a medication adherence packaging program, where patients are contacted two weeks prior to their monthly medication pickup or delivery date. The HRA consisted of 16 items and included questions on height, weight, current health conditions, safe housing, financial stability, transportation needs, depression screening, alcohol, drug and tobacco use, medication adherence, recent health screenings, lifestyle goals, and access to a primary care provider.

The installation stage included training of pharmacy staff and testing how to most efficiently administer the HRA. Pharmacy staff were trained on how to administer the HRA, what to do if patients needed immediate assistance, how to provide referrals, and the documentation of responses. A document describing the health plan’s services and local resources (e.g., counseling services and transportation assistance) was created for pharmacy staff to utilize and to provide to patients as needed. The HRA was tested on a sample of health plan patients to assess the question order, time needed to administer the HRA, and patient engagement. As a result of this test, HRA questions were reordered to improve the flow of the conversation and a decision was made to administer the questions over two separate patient encounters to reduce the amount of time each patient had to spend responding to questions.

The project team utilized the preparatory work from the exploration and installation stages to inform the activities of the initial implementation stage. The HRA was incorporated into the medication synchronization and medication adherence packaging ABM models at the pharmacy. A list of eligible patients was generated from the pharmacy’s prescription software. From the list, a schedule was created based on the patient’s expected pickup or delivery date. To ensure no patients would be missed for the program, the project team leader assigned patients to the pharmacy staff daily and provided support when necessary. Staff were assigned patients they typically managed as part of their ABM activities to leverage their established relationships. At least three attempts were made to reach eligible patients. When patients were unable to be reached, the pharmacy staff attempted to find alternate phone numbers by looking at electronic prescriptions for additional phone numbers and reaching out to the patient’s doctor when necessary. During the HRA, pharmacy staff assessed patient responses and identified patient eligibility for enhanced patient care pharmacy services. For example, if patients affirmatively responded to the question about tobacco use, they were immediately asked about their readiness to quit. If interested, patients could opt-in to follow-up for smoking cessation with a pharmacist. Patient responses to the HRA were recorded in a standardized patient care documentation platform, the Pharmacist eCare plan [15]. Responses were also reported directly to the health plan provider via a secure website provided to the pharmacy staff. When pharmacy technicians completed the HRA interviews, the team leader reviewed patient responses to ensure that no additional follow-up was needed. The initial implementation occurred over two months (November–December) in 2020.

### 2.2. Project Participants and Data Collection

All adult patients of the pharmacy who were members of the participating health plan and were also participants in the pharmacy’s medication synchronization or adherence packaging programs were eligible for inclusion in the project. HRA results were compiled from the pharmacy’s documentation platform. Additional data points identified through the HRA, including the time taken to complete the HRA and new service opportunities (e.g., smoking cessation and vaccinations), were also recorded. Descriptive statistics were utilized to characterize the results. This project was considered to be a program evaluation and was not reviewed as human subject research by the University of Pittsburgh’s Institutional Review Board.

## 3. Results

Forty-nine patients were identified and contacted by the pharmacy team as part of the pilot project. Of these, 38 (77.6%) successfully completed the HRA. The mean patient age was 46.5 ± 15 years and 26 (68.4%) were female. The median time for the pharmacy staff to complete the HRA was 19 min (range, 11–59 min).

**Figure 1:**
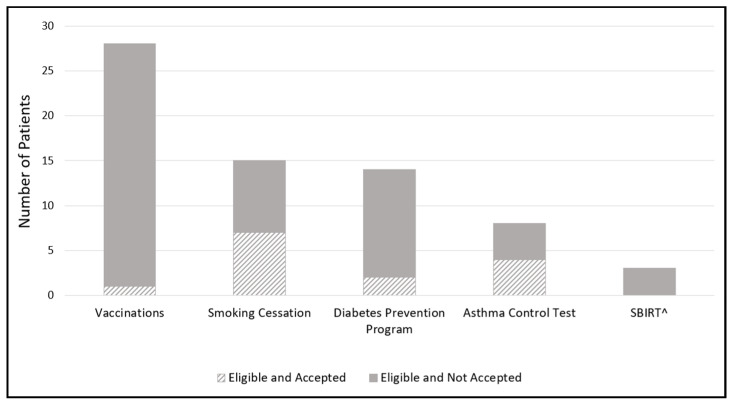
Number of patients eligible for pharmacy patient care services. (SBIRT^ = Screening, Brief Intervention, and Referral to Treatment for individuals with substance use disorders or those at risk for developing a substance use disorder.)

**Figure 2:**
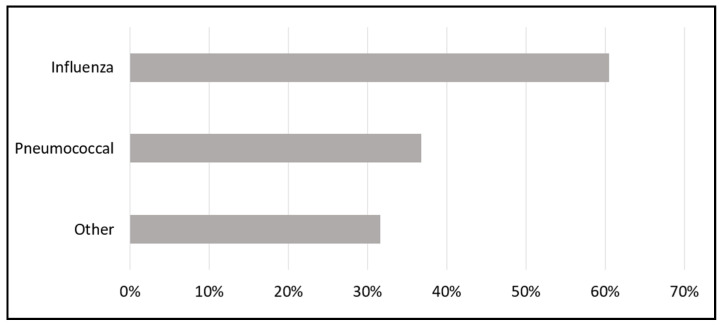
Percentage of patients with vaccination opportunities.

Pharmacy staff assessed patient responses to the HRA and identified opportunities for enhanced patient care pharmacy services. Vaccinations, smoking cessation, and diabetes prevention programs were the most commonly identified service opportunities (Figure 1). The pharmacy identified 28 (73.7%) patients with a total of 49 vaccination opportunities: 12 patients (32%) qualified for one, 11 (29%) qualified for two, and five (13%) qualified for three vaccinations. Influenza vaccination opportunities (23 of 38 patients) were most commonly identified (Figure 2).

Of the 15 patients who were regular tobacco users, 7 accepted the referral for smoking cessation. Two of fourteen patients that were eligible for the Diabetes Prevention Program (DPP) accepted the referral. DPP eligibility included patients that had an official diagnosis of pre-diabetes, those with a high risk of diabetes according to the American Diabetes Association’s risk assessment test, or those that were considered obese based on body mass index (BMI) [16]. Of the eight patients with asthma, four (50%) accepted assessment of their asthma control during the HRA using the asthma control test [17]. Based on the ACT results, one patient was referred to their physician for further assessment. Finally, three patients were identified as eligible for Screening, Brief Intervention and Referral to Treatment (SBIRT), a comprehensive approach for intervention and treatment services for individuals with substance use disorders [18].

## 4. Discussion

The goal of this pilot project was to implement an HRA for a health plan at a single independent community pharmacy and identify pharmacy-patient-care service opportunities through the assessment. The results demonstrated that approximately 78% of patients completed the HRA over the course of two pharmacy-patient encounters. HRA completion rates continue to be a challenge for health plan providers, with average HRA completion rates within 60 days of plan enrollment reported at approximately 39% [19]. Health plan providers often incentivize members and their health care providers to complete HRAs, although financial incentives have not been reported to be significant motivators for either group [5,20]. Zhang and colleagues emphasize the role of front-line clinicians, such as primary care providers, in improving completion rates for HRAs [20]. Accordingly, one state Medicaid plan provider reported a 49.3% HRA completion rate with recommendations from their primary care provider listed as the most common reason for completing an HRA by enrollees [5]. Although our project had a small sample size, our HRA completion rate was excellent for a Medicaid population that is often difficult to reach. Pharmacists are front-line clinicians with frequent access to patients. One factor that could have impacted our HRA completion rate success was the access the pharmacy had to this patient population through the ABM. Patients enrolled in the pharmacy’s medication synchronization or adherence packaging ABM programs are contacted monthly. The frequency and nature of these encounters, where pharmacy staff routinely inquire about patients’ medication use, may contribute to earning their trust over time [8]. 

Since independent community pharmacies usually have limited staffing resources, it may be difficult for some to allocate staff time for HRAs [21]. In this pilot project, it took approximately 19 min for our pharmacy staff to complete each HRA. The CDC recommends that HRA questions be limited in scope and prioritized to the most pertinent questions to reduce completion time to under 20 min [2]. Although the pharmacy did not receive a financial incentive for conducting this pilot work, the goal was to demonstrate feasibility so that payment could be sought for future similar programs. Partnerships between health plan providers and community pharmacies should include a financial incentive for the pharmacy to complete the HRA due to the value provided to the plan. Additionally, this study demonstrated that HRA completion can lead to increased billing opportunities for reimbursable patient care activities. For example, we found vaccination gaps in approximately 75% of patients through the HRA. However, the 2-month timeframe of this pilot coupled with the program occurring during the first year of the COVID-19 pandemic did not enable us to realize the full financial potential for new vaccination opportunities. Fitzpatrick and colleagues conducted a retrospective study describing drug therapy problems identified in patients enrolled in an appointment-based model [22]. They found that vaccine recommendations were the second most commonly documented intervention. Programs such as these can provide new revenue opportunities for community pharmacies.

Another important aspect of the HRA is the inclusion of questions addressing social determinants of health (SDoH). SDoH, such as housing stability, food insecurity, education, language, and literacy, can have a significant impact on health and well-being [23]. Pharmacists can play a crucial role in identifying, assessing, and mitigating SDoH in their patients [11,24]. Addressing SDoH is also important for pharmacies since they can negatively impact patients’ medication-taking behaviors. For example, medication adherence is adversely impacted in patients who lack transportation to pick up their medications or who have a language barrier that impacts their understanding of how to take their medication. Furthermore, recent research has linked food and housing insecurity to medication nonadherence [25]. Patients would ultimately benefit if their pharmacy screened for SDoH and provided them with appropriate resources and referrals.

We utilized an implementation framework to guide us through planning and operationalizing this project. There has been increased interest in the use of implementation science to advance patient care delivery in pharmacy settings [26,27]. As community pharmacies move from a product to a service and patient care focus, the use of implementation science principles is recommended to ensure effectiveness and sustainability of these new services [28]. We believe that the use of an implementation framework fostered a more in-depth, systematic approach to the HRA project and contributed to its success.

There are several limitations to this work. This project was a small pilot study conducted at a single independent community pharmacy in Pennsylvania. Accordingly, the results may not be generalizable to other geographic regions or types of pharmacies. Furthermore, the pharmacy in this project had a large number of patients enrolled in their ABM who were accustomed to being contacted by pharmacy staff on a regular basis. Pharmacies that do not have strong ABM programs may not achieve similar results. Additionally, since patients were enrolled in the pharmacy’s ABM, this may have resulted in a sampling bias and the results may not reflect other pharmacy-patient populations [29]. A final limitation was the short timeframe for the pilot, which did not allow for robust program implementation and follow-up with patients to engage them in the identified service opportunities.

## 5. Conclusions

This pilot demonstrated the effective implementation of an HRA into the ABM workflow at an independent community pharmacy. The information gained through performing an HRA can lead to new pharmacy service opportunities that contribute to improved patient care and practice sustainability.

## Data Availability

Not applicable.
